# Androgen receptor amplification is concordant between circulating tumor cells and biopsies from men undergoing treatment for metastatic castration resistant prostate cancer

**DOI:** 10.18632/oncotarget.16169

**Published:** 2017-03-13

**Authors:** Jennifer Podolak, Kristi Eilers, Timothy Newby, Rachel Slottke, Erin Tucker, Susan B. Olson, Hui-Wen Lue, Jack Youngren, Rahul Aggarwal, Eric J. Small, Julie N. Graff, Joshi J. Alumkal, Tomasz M. Beer, George V. Thomas

**Affiliations:** ^1^ Knight Cancer Institute, Oregon Health and Science University, Portland, OR, USA; ^2^ Department of Pathology and Laboratory Medicine, Oregon Health and Science University, Portland, OR, USA; ^3^ School of Medicine, University of California, San Francisco, CA, USA

**Keywords:** prostate cancer, circulating tumor cells, androgen receptor, metastases, metastatic castration resistant prostate cancer

## Abstract

Increased *AR* activity has been shown to be preserved in spatially distinct metastatic tumors from the same patient suggesting the requirement for lineage-specific dependencies for metastatic castration resistant prostate cancer (mCRPC). Amplification of the *AR* gene is a common mechanism by which mCRPC increase *AR* activity. To determine whether *AR* amplification in circulating tumor cells (CTC) could complement metastatic tissue biopsies in men undergoing treatment for mCRPC, we developed a novel two-step assay to isolate CTCs and subsequently analyzed *AR* amplification status in CTCs and matched biopsy tissue from the same patient by fluorescence *in situ* hybridization (FISH). *AR* gene status in CTCs showed strong concordance with *AR* gene status in matched tissue samples in 24 of 25 patients (Correlation: 96%; Kappa: 0.83; Sensitivity: 100%, Specificity: 83%). Our work demonstrates that *AR* amplification is conserved between CTCs and biopsies and that CTCs can serve as non-invasive surrogate to document *AR* amplification in mCRPC.

## INTRODUCTION

AR signaling is the primary driver of prostate cancer, and subsequently, medical castration with androgen deprivation therapy (ADT) is the backbone of all treatments in men with metastatic prostate cancer [1–4]. However, these tumors invariably become resistant to ADT, with the emergence of CRPC, which is ultimately fatal. Increased *AR* signaling despite castrate levels of testosterone is responsible for CRPC in the majority of men. Importantly, the majority of spatially distinct metastases within individual patients with mCRPC continue to exhibit increased *AR* signaling [5]. Hematogenous dissemination of cancer cells is a critical step in the development of metastases. Consequently, CTCs in the bloodstream potentially link the primary tumor with their anatomically and chronologically distinct metastatic progenies [6, 7]. Therefore, our objective was to determine whether CTCs could serve as non-invasive surrogates for biopsies of metastatic tissues. Specifically, we determined whether men treated with more potent androgen signaling inhibitors (ASI) such as abiraterone and enzalutamide continue to exhibit higher androgen signaling activity [8–10]. One mechanism of increased *AR* signaling in CRPC is through amplification of the *AR* gene [5]. We developed a platform-agnostic protocol to capture CTCs and to measure *AR* amplification status in CTCs from men with mCRPC undergoing treatment, and we evaluated the concordance of *AR* amplification status with matched metastatic biopsies.

## RESULTS

### Validation of CTC capture protocol and *AR* FISH

**Figure 1:**
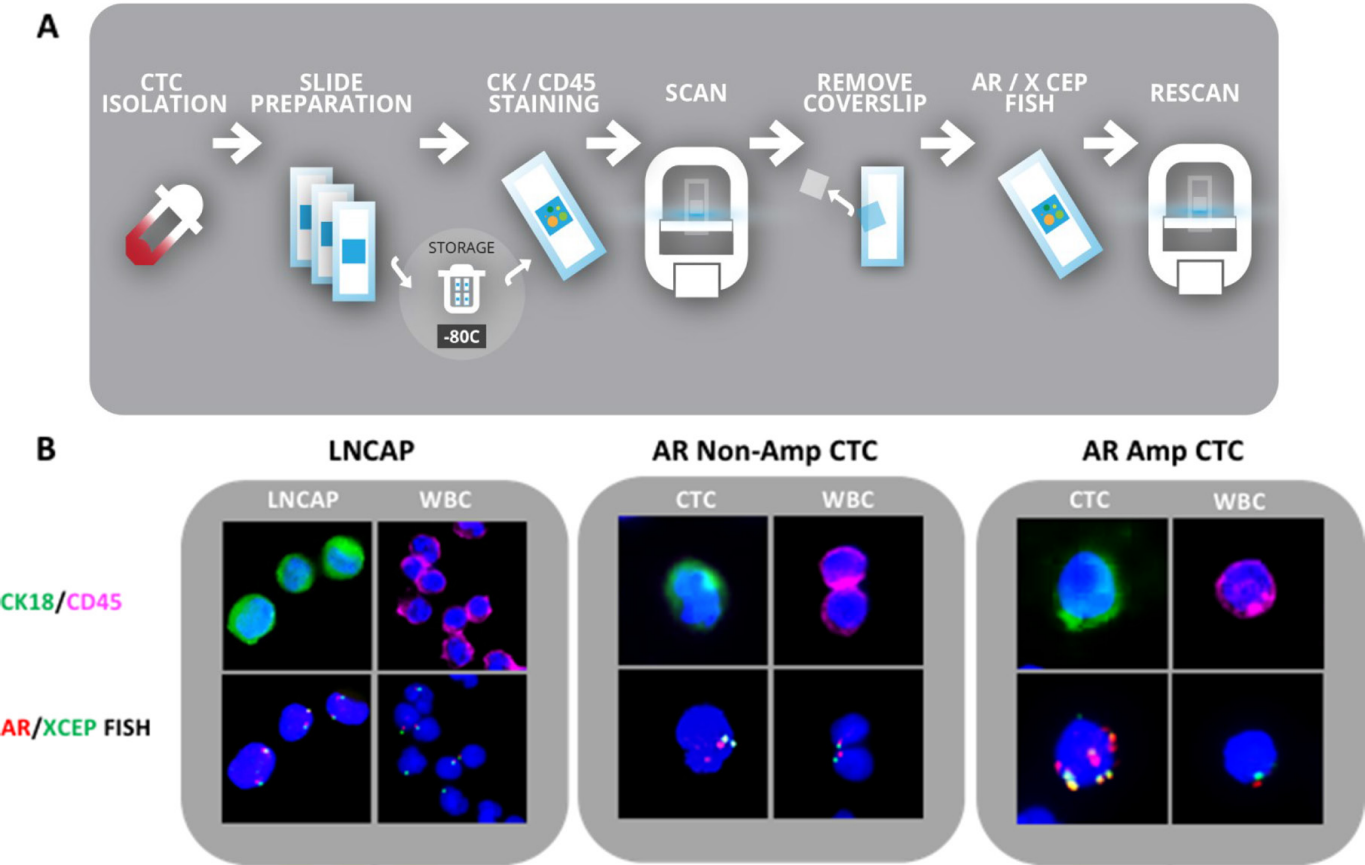
CTC capture and validation in LNCAP and mCRPC patients (**A**) Flowchart of CTC isolation, detection and molecular analysis workflow. (**B**) Left panel: LNCAP cells spiked into blood as a model for CTC capture and assay validation; Middle panel: Patient CTC without *AR* amplification; Right panel: Patient CTC with *AR* amplification. Upper panel immunofluorescence: Blue: dapi, Green: CK20, Pink: CD45. Lower panel: *AR* FISH analysis of cell identified in upper panel. Red: *AR* signal, green: *X CEP* control signal.

To demonstrate the validity of our CTC isolation method, we first spiked 10, 250, 500 and 1000 LNCAP cells into 15 mls of normal whole blood. The blood/cell mixture was diluted 1:1 with PBS. Fifteen mls of Ficoll Paque Plus (GE Healthcare #17-1440-02) was added to a 50 ml Leucosep filter tube (Greiner Bio-One #227290P) and the blood/cell mixture was added to Leucosep tube and spun at 1600 g for 20 min without brake. After centrifugation, the PBMC layer containing spiked LNCAP cells was removed using a transfer pipet and placed in a clean 15 ml conical and pelleted at 400 g for 4 min. Cell pellet was resuspended in 1 ml of donor blood set aside at the start of the assay. Fifty microliters of RosetteSep CTC enrichment cocktail (Stemcell # 15137C) was added to each sample and incubated at room temperature for 20 minutes. After incubation, 4 ml of Ficoll Paque Plus was added to a new 15 ml conical for each sample. Enriched blood samples were brought to a total volume of 6 ml with PBS and was gently layered onto the Ficoll layer. Samples were spun at 1200 rpm for 20 min without brake. Enriched cell layer was removed and placed in a new 15 ml conical. PBS was added to enriched cells to bring volume to 9 ml. One ml of red lysis buffer was added and conicals were incubated on a rocker for 15 min. Cell samples were pelleted at 400 g for 4 min and supernatant was decanted. Remaining cells were resuspended in 200 ul of 4% paraformaldehyde. The re-suspended cells were cytospun using a Thermo Scientific Cytospin 4 to create 2 slides per sample, 100 ul of suspension per slide. Funnel and slide assembly was constructed and 100 ul of cell suspension was added to each funnel. Slides were spun at 700 g for 3 min. Funnel and slide were disassembled; slides were allowed to air dry and then were stored at −80C for up to six months. To quantify the capture rate of this method, slides were stained with CK18 (green) and CD45 (pink) markers to differentiate epithelial cells from WBCs. Slides were scanned using a Panoramic Midi slide scanner (Perkin Elmer) and quantified by visual inspection. Our recovery assays were performed by spiking between 10 and 1000 LNCAP cells into donor blood samples. Multiple recovery assays were performed with an average yield of 38%. Despite the low recovery rate, we were able to capture cells in our samples that contained only 10 spiked cells providing confidence that CTC capture would be attainable in patient samples. After capturing full slide images using Panoramic MIDI scanner, the slides were reprocessed for *AR* gene status using FISH. Slides were re-imaged. LNCAP cells possessed 2 copies of *AR* and 2 copies *X CEP* gene and were identified by relocating the CK18 positive cells. White blood cells contained zero or one copy of *AR* gene and had zero or one copy of *X CEP* control. No white blood cells expressed greater than 1 copy of *X CEP* control, consistent with prior reported results [11]. (Figure 1).

### *AR* FISH status in CTCs revealed a strong concordance with *AR* status in matched tumor tissue

**Figure 2:**
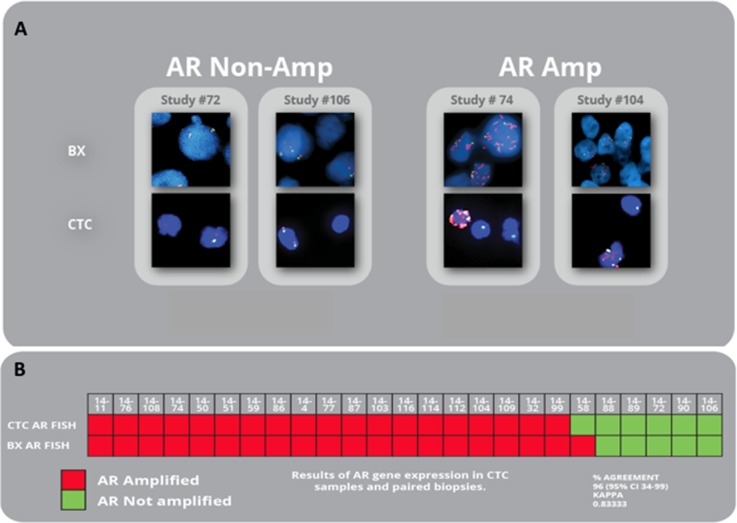
*AR* amplification in matched CTCs and biopsies from mCRPC patients (**A**) Representative images of paired CTC and biopsy patient samples without *AR* gene amplification (left) and paired patient samples expressing *AR* gene amplification (right). Red: *AR* signal; Green: Cep X control signal. (**B**) Results of *AR* gene expression in CTC samples and paired biopsies of all study subjects.

We compared *AR* gene status in CTC samples of 25 patients with CRPC status with the *AR* gene status of patient matched tumor tissue in a blinded approach (see Table 1 for clinical details). Our studies resulted in an observed kappa of 0.8837. The median interval between CTC samples and tissue sample was 3.72 days. The number of CTCs per patient sample ranged from 1–33. We were able to determine the *AR* gene status of all patient CTC samples and found that *AR* amplification occurred in 19 of 25 patients. Analysis of *AR* FISH status was performed and determined on all patient tissue samples. We were able to correlate all patient CTC samples with a matched tissue sample to determine *AR* gene amplification status. Nineteen patients had *AR* gene amplification status expressed in CTCs and tumor sample. Five patients had no *AR* gene amplification in CTC samples and tissue sample. One patient expressed *AR* gene amplification in CTCs, but not in the biopsy, perhaps representing heterogeneity among different metastatic sites on the same patient (Figure 2). Overall, CTC samples revealed the same *AR* gene status as the biopsy sample in 24 of 25 cases (Correlation:96%; Kappa: 0.83; Sensitivity: 100%, Specificity: 83%). Fifteen subjects had abiraterone or enzalutamide treatment prior to biopsy compared to ten subjects that did not receive these drugs. No statistical differences were observed between treatment and *AR* signaling activity. We also compared *AR* signaling to PSA, Gleason score and LDH and found no significant differences between treatment groups or those that responded to treatment and those that progressed.

**Table 1:** mCRPC patient characteristics and treatment history

Age at bx	
Median (IQR)	70 (57–83)
Gleason score, *n* (%)	
6	1 (4)
7	7 (28)
8-10	15 (60)
unk	2 (8)
Disease sites, *n* (%)	
bone	6 (24)
bone + lymph node	9 (36)
Bone + visceral	4 (16)
Bone + lymph node + visceral	1 (4)
Lymph node	4 (16)
Lymph node + visceral	1 (4)
ECOG, *n* (%)	
0	13 (52)
1	12(48)
Laboratory	
Hemoglobin (media, g/dL; IQR)	12.36 (9.6–14)
Low (< 13.5) n (%)	24 (96)
LDH (median, U/L; IQR)	213 (150–312)
Elevated (> 250), n (%)	4 (16)
Unknown	5 (20)
Albumin (median, g/L;IQR)	3.66 (3.0–4.2)
Low (< 35), n (%)	3 (12)
Normal, (%)	22 (88)
Current treatment, *n* (%)	
Abiraterone acetate	1 (4)
Enzalutamide	12 (48)
Radium	1 (4)
Docetaxel	
6 (24)	
Lupron	2 (8)
Cab/OGX	1 (4)
None	2 (8)
All treatments received, *n* (%)	
Abiraterone acetate	9 (36)
Enzalutamide	25 (100)
Docetaxel	1 (4)
Bicalutamide	22 (88)
LBH+Casodex	5 (20)
LHRH Agonist (Lupron, zoladex)	5 (20)
LHRH Antagonist (Degarelix)	25 (100)
Other	10 (40)

### CTCs reveal heterogeneous *AR* amplification in CRPC patients

**Figure 3:**
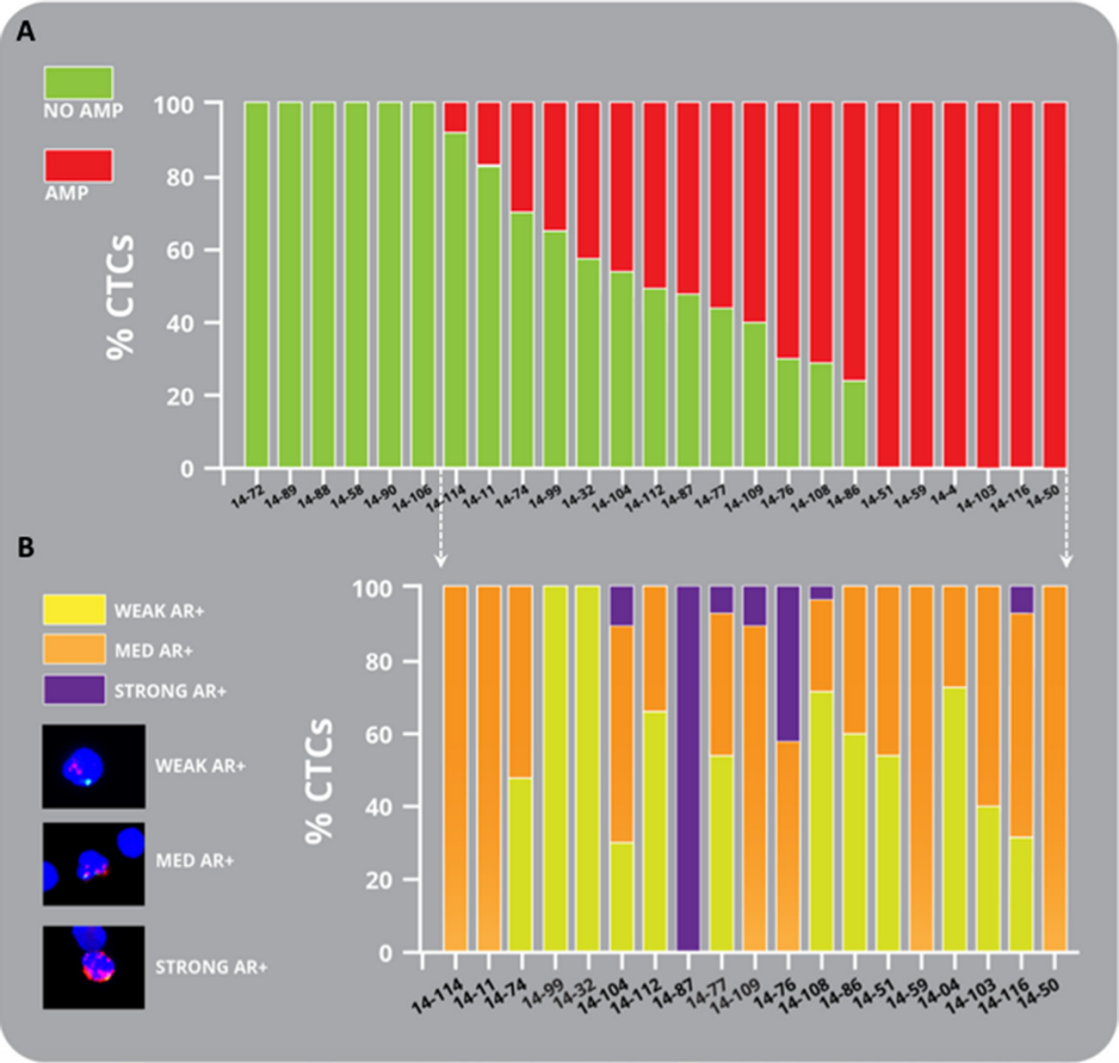
Heterogeneity of amplification in mCRPC patient CTCs (**A**) Bar graph representing the percentage of amplified CTCs (red) and non-amplified CTCs (green) found in mCRPC patients. (**B**) Intra-and interpatient heterogeneity of *AR* amplification in CTCs in mCRPC patients is shown graphically. Representative images are depicted of weak (yellow), medium (orange) and strong (purple) *AR* amplification found in mCRPC patient CTCs. See results for details.

We observed inter- and intra-patient heterogeneity for *AR* amplification (Figure 3, and Table 2). Thirteen of twenty-five patients (52%) exhibited a mixture of *AR* amplified positive and negative CTCs in the same sample. Using the FishQuant software, *AR* FISH scores were determined by the ratio of gene probe/control probe (G/C). For amplified, CTCs, we assigned G/C 2.5 as weak *AR* amplification, G/C > 2.5 and < 4 medium amplification and G/C > 4 defined strong *AR* amplification. Twelve of twenty patients (60%) exhibited different levels of *AR* amplification and points to multiple temporal and spatial tumor clones in CTCs from patients with CRPC, that may be the result of selective pressures from treatment or evolution of the cancer as it progresses from hormone sensitive to CRPC.

**Table 2:** *AR* amplification status in CTCs from mCRPC patients (related to Figure 3)

Study #	Serum PSA at bx	Total # CTCs	# WT CTC	# Amplified CTCs	% of Amplified CTCs
14–114	94.42	19	18	1	5.2
14–11	1.79	12	10	2	16.7
14–74	17.56	27	10	7	25.9
14–99	2137.3	18	13	5	27.8
14.32	1169	13	8	5	38.4
14–104	332.62	57	33	24	42.1
14–87	1859	2	1	1	50
14–77	11.82	48	23	25	52
14–109	425.28	551	22	29	56.9
14–76	4.54	40	11	29	72.5
14–108	10.57	79	21	58	73.4
14–86	120.06	26	6	20	76.9
14–72	37.06	20	20	0	0
14–89	6.18	18	18	0	0
14–88	173.5	6	6	0	0
14–58	48.92	4	4	0	0
14–90	148.39	4	4	0	0
14–106	371.76	1	1	0	0
14–51	109.93	2	0	2	100
14–59	215.9	8	0	8	100
14–4	250	3	0	3	100
14–103	8.33	5	0	5	100
14–116	20.9	40	0	40	100
14–50	41.83	1	0	1	100

### *AR* amplification in patients with sequential biopsies

**Figure 4:**
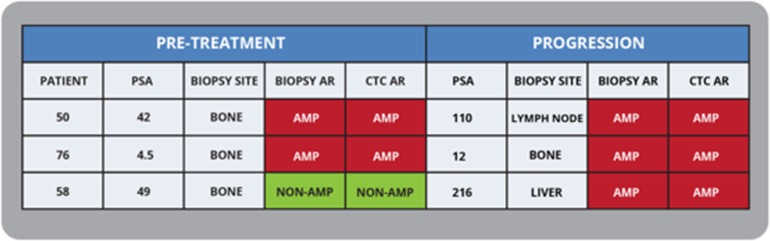
Documenting *AR* Amplification status with sequential CTC and biopsies in mCRPC patients Sequential CTC analysis correlated with baseline and progression biopsies, with two patients having *AR* amplification at both timepoints, and one patient developing *AR* amplification at time of progression.

We also investigated *AR* amplification in three patients with sequential samples collected before starting treatment and at progression (Figure 4). The CTC *AR* status matched the biopsy *AR* status in all three patients. Importantly, one patient *(ID# 58)* developed *AR* amplification at progression and this was picked up both in his metastatic liver biopsy and CTCs drawn at the same time.

## DISCUSSION

Hematogenous dissemination of cancer cells is a critical step in the development of metastases [12]. Consequently, CTCs in the bloodstream potentially link the primary tumor with their anatomically and chronologically distinct metastatic progenies. Patients with higher numbers of CTCs in their bloodstream do worse than those with none, and this is especially true for prostate cancer [13]. This then has been the basis of CTC capture devices, which were developed to enumerate the number of CTCs in drawn blood [14]. Our method, which can be performed in either research or clinical setting uses a combination of red cell lysis and WBC depletion to capture CTCs which can be then characterized by any surface marker and identified through an automated scanning imaging platform.

Circulating tumor cells offer a non-invasive method of determining the biological status of individual tumors, treatment evaluation and progression. The shedding of CTCs from the primary tumor is known to play a role in mCRPC. Studies have shown that the number of CTCs decreases with response to treatment and increases with disease progression. The study of genomic makeup of CTCs has been vastly increasing to include genomic and RNA profiling to identify of key drivers in CRPC. Recent studies have included *PTEN*, *ERG* and *AR* gene status and mRNA profiling in single circulating tumor cells [11, 15]. Molecular profiling of CTCs proves difficult due to low cell number and the high cost of assay platforms and routine testing, however, the biological information that can be derived from an individual's CTC profile may lead to improved individualized treatment.

Since *AR* amplification is a major driver of CRPC and persists despite newer Androgen Signaling Inhibitors such as abiraterone and enzalutamide, we evaluated *AR* amplification status by FISH. Simultaneously, we collected metastatic CRPC biopsies and performed *AR* FISH on FFPE tissues. *AR* gene status detected in CTCs showed strong concordance with *AR* gene status in matched tissue samples in 24 of 25 patients (96%).

Intriguingly, this study through our ability to identify and quantify single CTCs revealed that there is striking intra-patient heterogeneity in *AR* amplification status, both binary (i.e. amplified and non-amplified CTCs in the same patient) and the degree of amplification (low to high number of *AR* gene copies). This may reflect selective pressures brought on by different treatments and which may ultimately define the viability of metastatic clones. Alternatively, these CTCs may represent tumor cells shed from different metastatic sites and therefore reflect tumor heterogeneity. However, despite treatment with more potent androgen signaling inhibitors, *AR* amplification continues to function as a potential resistance mechanism. Accordingly, the opportunity to perform serial blood draws for CTCs can be used to monitor the emergence of *AR* amplification.

In summary, our results indicate the potential to expand CTC research through use of a simple, low cost assay to detect protein and gene expression in CTCs. Our research has shown that *AR* gene status can be accurately determined in CPRC patients using a non-invasive procedure that utilizes peripheral blood.

## MATERIALS AND METHODS

### Patient selection

Patients treated at Oregon Health & Science University with mCRPC were recruited under an institutional review board-approved protocol with informed consent. Specifically, men undergoing pretreatment and disease progression tumor biopsies for mCRPC were recruited under the SU2C/PCF/AACR West Coast Cancer Dream Team biopsy program. This study focuses on the patients recruited between Jan 2014 and June 2015. During that timeframe, 29 men were recruited. Here we report on the results of 25 of those men. Patients were selected based on having a tumor biopsy performed. CTC *AR* amplification results were blindly compared to the current gold standard tumor biopsy result for *AR* gene status. (R1 Q1) Four men were eliminated from the results because a paired tumor biopsy and CTC sample were not attained for various reasons. Two subjects were not tested for *AR* amplification in their tumor biopsy, one sample failed the tumor biopsy *AR* amplification assay and one sample failed the CTC *AR* amplification assay. Fifteen mls of blood for CTC studies were drawn for analysis at time of biopsy. Complete patient demographics are shown in Table 1.

### Protocol for isolation of CTCs from patient blood samples

Please see results section for detailed protocol. Blood samples were collected using two EDTA collection tubes. Blood was processed within 24 hours of collection in accordance with the above described protocol. Please see Figure 1A for flowchart.

### Immunofluorescent staining of patient samples

Immunofluorescent staining was performed on CTC samples. Slides were removed from −80C storage and allowed to reach room temperature. Once slides were room temperature they were placed in PBS. Within 20 minutes, permeabilization was performed for 20 minutes using cold methanol stored at −20C. Slides were rinsed in PBS. Nonspecific binding was blocked using Dako protein block (Dako X090930) for 30 minutes.

Primary antibodies were stained sequentially. First, CK18 1:100 ( Santa Cruz Bio # sc-31700) was incubated for 1 hour at room temperature. Slides were rinsed in PBS 2 × 5 minutes. Secondary antibody Alexa 488 (Life Tech A11034) 1:1000 was diluted in PBS and was incubated at room temperature for 30 minutes. Slides were washed in PBS for 5 minutes. Slides were then incubated for one hour at room temperature with anti- CD45 1:00 (Abcam #ab8216). Slides were rinsed in PBS for 2 × 5 min. Alexa 647 (Life Tech #A31571) 1:1000 diluted in PBS was incubated for 30 minutes at room temperature. Slides were rinsed in PBS for 5 minutes. Slides were air dried and immediately coverslipped using mounting media with Dapi (Vector #H-1200). Slides were immediately scanned or stored at 4C overnight and scanned the next day using Perkin Elmer Midi scanner. Slide image was visually reviewed to determine the number of positive CK18 negative CD45 cells. Following completion of scanning and image review, coverslips were removed from slides by soaking slides in PBS to allow coverslips to float off. Slides were then immediately processed for *AR* FISH probe.

### Fluorescent *in situ* hybridization (FISH)

A two color FISH assay was performed using Spectrum Orange *AR* (Xq12) probe (Vysis #30-190040) and Spectrum Green labeled ChrX centromere (Xp11.1-q11.1) *X CEP* (Vysis# 32-112023). Patient slides were permeabilized at 80C for 5 min in a 2xSCC/formamide solution. Slides were rinsed in PBS and dehydrated through a series of alcohols. Probes were prepared according to manufacturer's instructions and denatured at 72C for 5 min and hybridized overnight at 32C using Vysis hybridizer. The next day slides were washed in 2xSCC +0.1% tween solution at 42C for 2 min then rinsed in room temp PBS, dehydrated and coverslipped using Dako mounting media with Dapi (# H-1200). CTCs were identified by cells that expressed 2 or more copies of *AR* and *X CEP* probe, positive CK18 expression and negative CD45 expression. White blood cells were identified as having 1 copy of *AR* and 1 copy of *X CEP*, negative CK 18 expression and positive CD45 expression.

### Automated CTC *AR* FISH quantification

Slides were scanned on a 3D Histech Panoramic scanner. Fluorescent *in situ* hybridization signals were assessed using FishQuant software. *AR* amplification was considered to be present when the *AR* gene to X chromosome ratio was greater than *2*. Some leukocytes remained following isolation process and were used as internal controls.

### Metastatic CRPC biopsies

Five μm FFPE tissue sections were mounted on positively charged glass slides. One section was stained with H&E and examined by a pathologist, followed by marking of tumor region(s). Slides for FISH were baked in a dry oven at 60 degrees C for 2 hours. They were then cooled briefly and loaded into a Vysis VP2000 slide processor and were then deparaffinized and pretreated. Slides were dried and 5–10 μl of an androgen receptor (AR)/ X centromere probe solution were added to each slide. Slides were covered with glass coverslips, sealed with rubber cement, and placed into a Vysis Thermobrite machine for denaturation/renaturation. Coverslips were soaked off, slides post-washed and air dried before mounting with DAPI I using new glass coverslips. 100 cells were scored for the number of *AR* and centromere signals. Subsequently, fifty interphase cells were scored. A ratio of AR:X centromere signals > 2.0 was considered amplified.

### Statistical analysis

Positive predictive value, specificity and sensitivity were calculated based on the classification of patients for *AR* status from CTCs and archival tissue. Twenty-nine patients had matched biopsies and CTCs. Of these, 1 biopsy failed *AR* FISH testing, 1 CTC sample failed *AR* FISH testing, and 2 biopsies were inadequate for *AR* FISH. Subsequently, 25 patients were included in the final analysis as they had conclusive FISH results in both CTCs and tumor tissue. Descriptive statistical analyses, including Cohen's kappa testing were performed using GraphPad Prism software.
